# Toward Mass Drug Administration Stopping Criteria for *Schistosoma mansoni* Control Programs

**DOI:** 10.4269/ajtmh.19-0011

**Published:** 2019-02-04

**Authors:** W. Evan Secor

**Affiliations:** Division of Parasitic Diseases and Malaria, Centers for Disease Control and Prevention, Atlanta, Georgia

National control programs for the preventive chemotherapy of neglected tropical diseases (NTDs) use mass drug administration (MDA) to reduce infection levels in communities and rely on diagnostic tests to monitor program progress and inform interventions. Control programs for *Schistosoma mansoni* have conventionally employed stool examinations using the Kato–Katz method for program monitoring. Limitations of the Kato–Katz technique include the substantial time and effort required to make and read slides, its poor sensitivity at low levels of infection, and the occasional unwillingness of participants to provide stool samples. Nevertheless, it is widely available and has been the most relied-on technique for evaluating the prevalence of intestinal schistosomiasis for decades. Therefore, it is the method recommended by the current WHO guidelines for *S. mansoni* control programs.

The introduction of a commercially available point-of-care (POC) test for circulating cathodic antigen (CCA) provides the promise of a more field-friendly rapid test for monitoring *S. mansoni* control programs. Like the Kato–Katz method, it can distinguish active from former infections and rapidly becomes negative, following successful treatment. Unlike Kato–Katz, it is a measure of worms rather than eggs, as CCA is produced in the parasite’s blind gut and regurgitated into the bloodstream. The antigen is then cleared by the kidneys and excreted in host urine. The POC-CCA has better sensitivity than Kato–Katz for light infections. It is also easier to obtain urine than stool from individuals being monitored, and despite being a commercial product, the POC-CCA is comparable in cost to Kato–Katz when all expenses are taken into consideration.^1^ However, as with the introduction of any new test, especially if there are differences in sensitivity or specificity compared with the existing test, new cutoffs must be established for proper program use.

Two recent articles now provide direction for program decision-making using the POC-CCA.^2,3^ The first, published in this issue of the *American Journal of Tropical Medicine and Hygiene*, sought to address the question of the “true” status of individuals who test positive by POC-CCA but negative by Kato–Katz—do these results represent a false-positive result by POC-CCA or the poor sensitivity of the Kato–Katz for light intensity infections? Daily fluctuations in egg excretion may explain the discordant results. The investigators therefore identified 45 Egyptian children between 8 and 13 years of age who were POC-CCA positive but egg negative and undertook the prodigious challenge of obtaining daily stool and urine samples from each child for a full month. Stool samples were examined by multiple Kato–Katz slides and the miracidial hatching test that uses a much larger quantity of stool, which better addresses the irregular distribution of schistosome eggs in feces. Of the 1,338 stool samples collected from this cohort, only one (0.07%) was egg positive. The sensitivity of the egg detection methods was validated by the inclusion of low-intensity positive controls in the latter half of the study. Furthermore, the urine samples collected over the course of the study were regularly positive (> 89% trace or +1 POC-CCA readings) according to the manufacturer’s criteria. The consistently antigen-positive, egg-negative results could reflect either imperfect specificity of the POC-CCA or the presence of single-sex or non–egg-producing infections in the studied children. Additional studies testing for the presence of anti-schistosome antibodies or changes in POC-CCA results after treatment will be needed to differentiate these two possibilities, but in either scenario, such individuals are unlikely to be shedding eggs into the environment that would perpetuate the schistosomiasis transmission cycle.^2^

The results from Haggag et al. suggest that egg output by a population can approach zero, whereas some individuals consistently remain trace or 1+ positive by POC-CCA. Defining the level at which this occurs may provide control programs with a target for MDA stopping decisions. For example, when all the children (*n* = 2,119) in the districts from which the study group were enrolled are considered, the average (±SD) Kato–Katz result was 0.7% ± 0.6% positive and the mean prevalence for POC-CCA was 9.4% ± 1.6%.^4^ A similar result was reported in a second recent publication.^3^ Bärenbold et al. incorporated the data from 30 studies that had individual-level Kato–Katz and POC-CCA results and developed mathematical models to define the corresponding results between the two tests. Along with determining other Kato–Katz/POC-CCA equivalents, the authors concluded that areas with 10% positivity by POC-CCA had approximately a 1% prevalence by Kato–Katz.^3^ Together, these studies suggest that communities with ≤ 10% trace or 1+ results by POC-CCA may be approaching interruption of *S. mansoni* transmission.

It is imperative that further studies beconducted to confirm whether the 10% trace or 1+ POC-CCA result is an appropriate MDA stopping target for *S. mansoni* control programs, but establishing tentative targets facilitates the study design and sample size calculations that will be needed to confirm or modify this value. No previous MDA stopping criteria for schistosomiasis have been defined using the Kato–Katz method. Without a defined target, it is challenging to develop strategies to achieve the MDA stopping goal. It is well recognized that MDA alone will not be sufficient to interrupt schistosomiasis transmission, but having a target will facilitate operational research to determine what adjunct intervention(s) (e.g., clean water, sanitation, snail control, or behavioral change) can most effectively complement MDA to achieve elimination.

Definition of criteria for making MDA decisions based on POC-CCA results may have additional implications for control programs. Schistosomiasis is a very focal disease, making it more difficult to make accurate treatment decisions at the district level compared with other NTDs. By conducting POC-CCA testing during MDA visits, it may be possible to obtain assessment data with greater granularity in a cost-effective manner. Most of the costs associated with MDA or assessments are related to transportation and personnel expenditures.^1^ For a relatively small incremental expenditure (e.g., the cost of the test cassettes and perhaps one more field worker), urine samples could be collected from a subset of MDA recipients while drug is being distributed and POC-CCA results could be used either for treatment decisions in real time or for tracking trends. Additional operational research will be needed to define how granular the sampling should be, how often data should be collected, and how many persons should be tested to obtain data for accurate program decision-making, but the studies by Haggag, Bärenbold, and their coauthors provide important information toward realizing this goal.

The findings and conclusions in this report are those of the authors and do not necessarily represent the official position of the Centers for Disease Control and Prevention.
